# The Association between Sampling and Survival in Patients with Pancreatic Ductal Adenocarcinoma Who Received Neoadjuvant Therapy and Pancreaticoduodenectomy

**DOI:** 10.3390/cancers16193312

**Published:** 2024-09-27

**Authors:** Mehran Taherian, Matthew H. G. Katz, Laura R. Prakash, Dongguang Wei, Yi Tat Tong, Zongshan Lai, Deyali Chatterjee, Hua Wang, Michael Kim, Ching-Wei D. Tzeng, Naruhiko Ikoma, Robert A. Wolff, Dan Zhao, Eugene J. Koay, Anirban Maitra, Huamin Wang

**Affiliations:** 1 Department of Pathology, The University of Texas MD Anderson Cancer Center, Houston, TX 77030, USA; mehran.x.taherian@kp.org (M.T.); donwei@ucdavis.edu (D.W.); to1283@gmail.com (Y.T.T.); zongshan99@gmail.com (Z.L.); dchatterjee@mdanderson.org (D.C.);; 2 Department of Surgical Oncology, The University of Texas MD Anderson Cancer Center, Houston, TX 77030, USA; mhgkatz@mdanderson.org (M.H.G.K.); lrprakash@mdanderson.org (L.R.P.); mkim@mdanderson.org (M.K.); cdtzeng@mdanderson.org (C.-W.D.T.); nikoma@mdanderson.org (N.I.); 3 Department of Gastrointestinal Medical Oncology, The University of Texas MD Anderson Cancer Center, Houston, TX 77030, USA; huawang@mdanderson.org (H.W.); rwolff@mdanderson.org (R.A.W.); dzhao3@mdanderson.org (D.Z.); 4 Department of Radiation Oncology, The University of Texas MD Anderson Cancer Center, Houston, TX 77030, USA; ekoay@mdanderson.org

**Keywords:** sampling, pancreatic cancer, neoadjuvant therapy, survival

## Abstract

**Simple Summary:**

We examined the association of the entire submission of the tumor (ESOT) and the entire submission of the pancreas (ESOP) with clinicopathologic features and survival in 627 pancreatic cancer patients who received neoadjuvant therapy (NAT). We demonstrated that both ESOT and ESOP were associated with lower ypT, less frequent perineural invasion, and better tumor response. Both ESOT and ESOP were associated with less frequent recurrence/metastasis, better disease-free survival (DFS), and overall survival (OS) in the overall study population. ESOP was associated with better DFS and OS in patients with ypT0/ypT1 or ypN0 tumors and better OS in patients with complete or near-complete response. ESOT was associated with better OS in patients with ypT0/ypT1 or ypN0 tumors. Both ESOT and ESOP were independent prognostic factors for OS in multivariate survival analyses. Therefore, ESOP and ESOT are associated with the prognosis of PDAC patients with complete or near-complete response and a ypT0/ypT1 tumor after NAT.

**Abstract:**

Adequate sampling is essential to an accurate pathologic evaluation of pancreatectomy specimens resected for pancreatic ductal adenocarcinoma (PDAC) after neoadjuvant therapy (NAT). However, limited data are available for the association between the sampling and survival in these patients. We examined the association of the entire submission of the tumor (ESOT) and the entire submission of the pancreas (ESOP) with disease-free survival (DFS) and overall survival (OS), as well as their correlations with clinicopathologic features, for 627 patients with PDAC who received NAT and pancreaticoduodenectomy. We demonstrated that both ESOT and ESOP were associated with lower ypT, less frequent perineural invasion, and better tumor response (*p* < 0.05). ESOP was also associated with a smaller tumor size (*p* < 0.001), more lymph nodes (*p* < 0.001), a lower ypN stage (*p* < 0.001), better differentiation (*p* = 0.02), and less frequent lymphovascular invasion (*p* = 0.009). However, since ESOP and ESOT were primarily conducted for cases with no grossly identifiable tumor or minimal residual carcinoma in initial sections, potential bias cannot be excluded. Both ESOT and ESOP were associated with less frequent recurrence/metastasis and better DFS and OS (*p* < 0.05) in the overall study population. ESOP was associated with better DFS and better OS in patients with ypT0/ypT1 or ypN0 tumors and better OS in patients with complete or near-complete response (*p* < 0.05). ESOT was associated with better OS in patients with ypT0/ypT1 or ypN0 tumors (*p* < 0.05). Both ESOT and ESOP were independent prognostic factors for OS according to multivariate survival analyses. Therefore, accurate pathologic evaluation using ESOP and ESOT is associated with the prognosis in PDAC patients with complete or near-complete pathologic response and ypT0/ypT1 tumor after NAT.

## 1. Introduction

Pancreatic ductal adenocarcinoma (PDAC), the most common type of pancreatic cancer, is one of the most fatal human cancers with an average 5-year survival rate of 10.8% [1]. Neoadjuvant therapy (NAT) is increasingly used in the treatment of patients with potentially resectable PDAC [2], and it has been shown to improve disease-free survival (DFS) and overall survival (OS) in patients with borderline resectable PDAC [3,4,5,6]. The current National Comprehensive Cancer Network (NCCN) guidelines and the guidelines from the American Society of Clinical Oncology (ASCO) recommend NAT as the standard of care for patients with borderline resectable PDAC, high-risk patients with resectable disease, and selected patients with locally advanced disease [7,8].

Previous studies have shown that the systematic histopathologic assessment of post-therapy pancreatectomy specimens resected for PDAC is important not only for prognostication but also for the post-operative management of these patients [9,10,11,12]. Multiple pathologic parameters, including the post-treatment tumor stage (ypT), lymph node stage (ypN), tumor response grading to NAT, integrated pathologic score, vascular invasion, perineural invasion, and margin status, are important prognosticators in patients with PDAC who received NAT and pancreatectomy [9,10,11,12,13,14,15,16,17,18,19,20,21,22,23,24,25,26].

The pathologic examination of post-therapy pancreatectomy specimens is complex and challenging because NAT often induces a heterogeneous response in different areas of the tumor and extensive fibrosis in both the tumor and adjacent non-neoplastic pancreas [14]. Therapy-induced fibrosis and preexisting fibrosis related to obstructive changes in the adjacent pancreatic tissue obscure the tumor boundaries, which makes the identification of a tumor and tumor-size measurements extremely difficult [14,27,28]. In addition, microscopic tumor invasion beyond the grossly identified tumor areas into the adjacent pancreas, peripancreatic soft tissue, or organs is common for patients with PDAC who underwent surgical resection with or without receiving NAT. For patients whose tumors have a marked treatment response and no grossly identifiable tumor, extensive sampling of the pancreas and adjacent tissue often reveals a significant amount of residual carcinoma, which cannot be accurately identified through a gross examination, or microscopic foci of viable carcinoma [14]. Therefore, the adequate sampling of the tumor and adjacent pancreatic tissue is important for the accurate pathologic evaluation and staging of post-therapy pancreatectomy specimens. The recent recommendations from the Pancreatobiliary Pathology Society (PBPS) recommended systemic and standardized examination and reporting for pancreatectomy specimens resected after NAT [12]. The PBPS recommended that smaller tumors ≤2.0 cm should be entirely submitted, and the larger tumors may be sampled generously with at least 2 sections per centimeter of the tumor. For cases with a marked pathologic response, which have either no grossly visible tumor or only microscopic residual tumor in the initial representative sections of the tumor, the PBPS and other studies emphasized the submission of the entire pancreas or rigorous, extensive sampling of the pancreas and peripancreatic soft tissue, common bile duct, ampulla of Vater, and duodenum adjacent to the pancreas [12,29]. However, limited data are available on the association between the sampling of the tumor or pancreas of post-therapy pancreatectomy specimens and the clinical outcome and survival of patients with PDAC who received NAT. In this large, retrospective study, we examined the impact of the entire submission of the tumor and the pancreas in 627 patients with PDAC who received NAT. Our results demonstrated that the entire submission of the tumor and the pancreas through post-therapy pancreatectomy are important prognosticators for survival.

## 2. Materials and Methods

### 2.1. Study Population

Approval from the institutional review board was obtained, and a waiver of consent was granted for using patients’ clinical and pathologic information for research. Our study population consisted of 627 patients with PDAC who received NAT followed by pancreaticoduodenectomy (PD) at our institution between January 1999 and December 2019. There were 283 females (45.1%) and 344 males (54.9%) with a median age of 64.5 years (range: 30.3 to 85.4 years). Fifty-seven patients with PDAC who underwent distal pancreatectomy and 11 patients who underwent total pancreatectomy after being treated with NAT were excluded since the aim of this study was to focus on the impact of sampling PD specimens on survival. The pretreatment pathologic diagnosis of PDAC was confirmed in all cases by reviewing fine-needle aspiration cytology and/or biopsies.

### 2.2. Pathologic Evaluation of Pancreaticoduodenectomies

The PD specimens were grossly and microscopically evaluated and reported using a standardized protocol, which included the tumor location, size, tumor type, differentiation, tumor involvement of extra-pancreatic tissue/organ(s), histologic tumor response grade, presence or absence of lymphovascular or perineural invasion, number of positive and total lymph nodes, and margin status, etc. The pathology of all cases was re-reviewed. The uncinate margin was entirely submitted perpendicularly for a histologic examination in all cases. All cases had ≥12 lymph nodes to ensure accurate lymph-node (pN) staging. The grading of the extent of residual carcinoma in PD specimens was performed using the MD Anderson and the College of American Pathologists (CAP) grading schemes. The post-treatment pathologic staging was grouped according to the *American Joint Committee on Cancer (AJCC) Staging Manual*, 8th edition.

The information on sampling of the entire tumor and pancreas, and the number of blocks from the pancreas, were collected based on the review of the gross description and section codes from the pathology report of each case. Only cases for which the entire head of the pancreas, common bile duct, peripancreatic tissue, and ampulla of Vater were submitted for histologic examination were classified as an entire submission of the pancreas (ESOP).

### 2.3. Clinical and Follow-Up Data

Clinical and follow-up information, including age at diagnosis, gender, tumor resectability, neoadjuvant chemotherapy regimen, neoadjuvant radiation, type of surgery, the date and site of recurrence, and the date and cause of death, were retrieved from a prospectively maintained database for patients with pancreatic cancer. All clinical and follow-up information was verified by reviewing patients’ medical records and, if necessary, the United States Social Security Index. Disease recurrence or metastasis was determined largely based on radiographic and clinical suspicion, as definite confirmatory biopsies were not routinely performed on all patients.

### 2.4. Statistical Analyses

The categorized clinicopathologic features were compared between groups with and without the entire submission of the tumor or pancreas using Chi-square analyses. Independent-sample T tests were used to compare continuous variables. Disease-free survival (DFS) was calculated from the date of PD to the date of the first recurrence after PD in patients with recurrence or to the last follow-up date in patients who had no recurrence. Overall survival (OS) was calculated from the date of diagnosis to the date of death or the date of last follow-up if death did not occur. The follow-up time ranged from 6.7 to 257.5 months with a median of 33.0 months in the overall study group. The Kaplan–Meier method and log-rank test were used to compare the group with entire submissions of the tumor or pancreas to the group without an entire submission of the tumor or pancreas. Multivariate Cox regression analyses were performed to predict the DFS and OS based on the entire submission of the tumor or pancreas combined with other clinicopathologic covariates. A statistical analysis was performed using the Statistical Package for Social Sciences software for Windows (Version 26, SPSS Inc., Chicago, IL, USA). A 2-sided significance level of 0.05 was used for all statistical analyses.

## 3. Results

### 3.1. Correlations of Entire Submission of the Tumor or Pancreas with Clinicopathologic Parameters

Among the 627 patients, 17 (2.7%), 205 (32.7%), 325 (51.8%), and 80 (12.8%) cases were ypT0, ypT1, ypT2, and ypT3, respectively, and 284 (45.3%), 223 (35.6%) and 120 (19.1%) cases were ypN0, ypN1, and ypN2, respectively. There were 94 patients with a microscopically positive margin (R1, 15%), and 533 (85%) had a negative resection margin (R0). CAP grade 0, 1, 2, and 3 responses were observed in 17 (2.7%), 75 (12.0%), 330 (52.6%), and 205 (32.7%) cases, respectively. MD Anderson grade 0, 1, and 2 responses were observed in 17 (2.7%), 75 (12.0%), and 535 (85.3%) cases, respectively. The number of blocks submitted from the tumor and pancreas (including tumor blocks) ranged from 3 to 73 (median: 21). Only 16 (2.6%) had ≤10 blocks submitted from the pancreas in our study population. The entire tumor and entire pancreas were submitted in 333 (53.1%) and 101 (16%) cases, respectively. 

The correlations between the entire submission of the tumor (ESOT) and the entire submission of the pancreas (ESOP) and clinicopathologic parameters are shown in Table 1. Both ESOT and ESOP were associated with lower ypT stages, better tumor responses using either the CAP or the MD Anderson grading scheme, and less frequent perineural invasion and recurrence/metastasis (*p* < 0.05). In addition, ESOP was also associated with better tumor differentiation, less frequent lymphovascular invasion, and lower ypN stages (*p* < 0.05). Compared to the group without ESOP, the ESOP group had smaller tumors (1.89 ± 1.82 cm vs. 2.78 ± 1.19 cm, *p* < 0.001) and more lymph nodes (29.7 ± 11.3 vs. 25.2 ± 9.8, *p* < 0.001), but there was no significant difference in the number of positive lymph nodes between these two groups (1.71 ± 4.0 vs. 2.00 ± 3.0, *p* = 0.40).

### 3.2. Correlation between Entire Submission of the Tumor or the Pancreas and Survival

**Figure 1:**
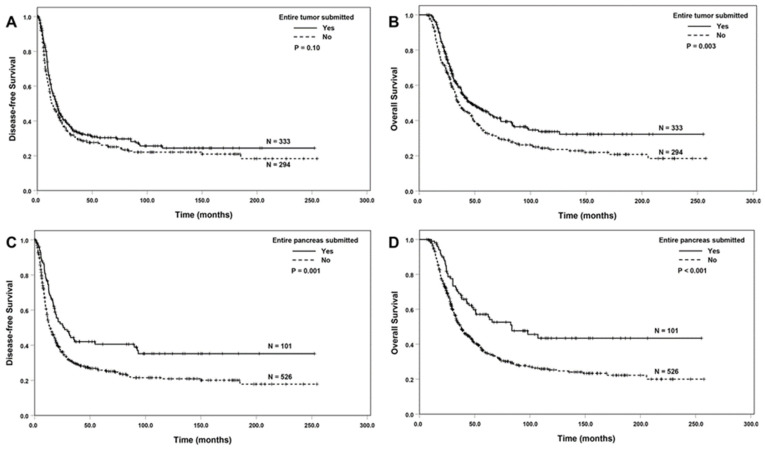
The Kaplan–Meier survival curves of disease-free survival and overall survival in the overall population (627 patients), comparing the group with ESOT (**A**,**B**) or ESOP (**C**,**D**) to the group without the submission of the tumor or the pancreas.

In the overall study group, ESOT was associated with better OS compared to the group without ESOT (median OS: 45.5 ± 6.1 months vs. 35.5 ± 3.0 months; *p* = 0.003), but not DFS (*p* = 0.10, Figure 1A,B). The median DFS and OS for the group with ESOP were 25.8 ± 5.9 months and 83.7 ± 20.7 months, respectively, compared to 13.9 ± 1.2 months (*p* = 0.001) and 37.8 ± 2.3 months (*p* < 0.001), respectively, for the group without ESOP (Figure 1C,D).

**Figure 2:**
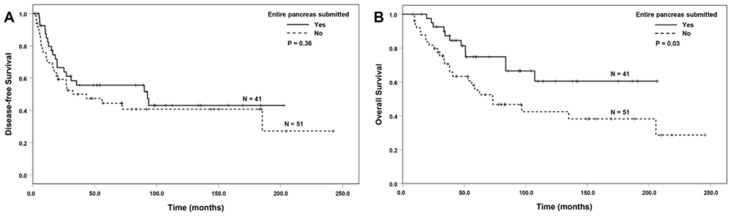
The Kaplan–Meier survival curves of disease-free survival and overall survival in patients with a complete or near-complete pathologic response (MD Anderson grade 0 or 1). There was no difference in disease-free survival between the group with ESOP and those without ESOP ((**A**), *p* = 0.36). ESOP was associated with better overall survival ((**B**), *p* = 0.03).

Among the patients who had a complete or near-complete pathologic response (MD Anderson grade 0 or 1), ESOP was associated with better OS compared to those without ESOP (*p* = 0.03), but there was no difference in DFS between the group with ESOP and those without ESOP (*p* = 0.36, Figure 2A,B). There were no differences in either DFS (*p* = 0.77) or OS (*p* = 0.28) between the group with ESOT and those without ESOT (*p* > 0.05). Among the patients with MD Anderson grade 2, CAP grade 2, or CAP grade 3 responses, no significant associations between ESOT or ESOP and either DFS or OS were observed (*p* > 0.05).

**Figure 3:**
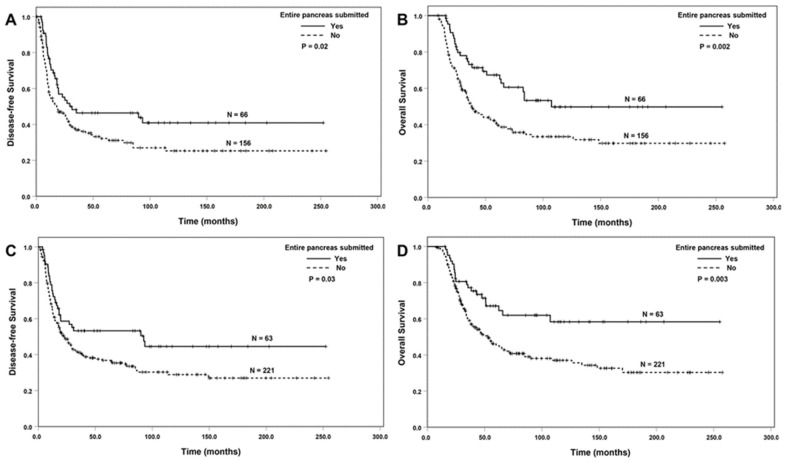
The Kaplan–Meier survival curves showing that ESOP was associated with better disease-free survival and overall survival compared to those without ESOP in patients with ypT0/ypT1 tumors (**A**,**B**) or ypN0 tumors (**C**,**D**).

**Figure 4:**
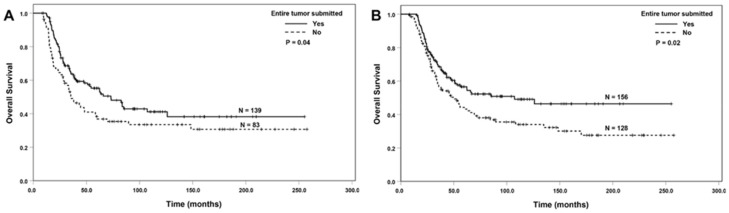
The Kaplan–Meier survival curves showing that ESOT was associated with better overall survival compared to those without ESOT in patients with ypT0/ypT1 tumors (**A**) and ypN0 tumors (**B**).

ESOP was associated with better DFS and OS among the patients with ypT0/T1 or ypN0 tumors (*p* < 0.05, Figure 3A–D), but not among the patients with ypT2/ypT3 or patients with positive lymph nodes (*p* > 0.05). ESOT was associated with better OS, but not DFS among the patients with ypT0/T1 or ypN0 tumors (*p* < 0.05, Figure 4A,B). There were no significant associations between ESOT and DFS or OS among the patients with a ypT2/ypT3 tumor or the patients with positive lymph nodes compared to the group without ESOT (*p* > 0.05).

### 3.3. Univariate and Multivariate Survival Analyses

To further examine the association between ESOT and ESOP and patient survival, we performed univariate and multivariate Cox regression analyses (Table 2, Table 3 and Table 4). We found that both ESOP [hazard ratio (HR): 0.72; 95% CI: 0.53–0.99; *p* = 0.04] and ESOT (HR: 0.80; 95% CI: 0.65–0.98; *p* = 0.03) were independent prognostic factors for OS, but not for DFS. In addition, ypN stage, tumor response grading, and tumor differentiation were also independent prognostic factors for both DFS and OS (*p* < 0.05, Table 2 and Table 3).

## 4. Discussion

The adequate sampling of the pancreatectomy specimens from patients with PDAC who received NAT is critical for the accurate histopathologic evaluation of tumor and lymph-node staging, the tumor response to NAT, and other pathologic parameters. However, limited data are available on the optimal sampling approach and the association of tumor and pancreas sampling with the clinical outcomes and survival. In this study, we retrospectively reviewed the association between the entire submission of the tumor and the entire submission of the pancreas and the clinicopathologic parameters and survival in a large cohort of 627 patients with PDAC who underwent PD after receiving NAT. We demonstrated for the first time that ESOT and ESOP are associated with lower ypT, less frequent perineural invasion, and better tumor response grade using either MD Anderson or the CAP grading system. ESOP was also associated with a lower ypN stage, better tumor differentiation and less frequent lymphovascular invasion, a smaller tumor size, and more lymph nodes. More importantly, we demonstrated that ESOT and ESOP were associated with less frequent recurrence/metastasis and better survival and were independent prognostic factors for OS. Our results provide much-needed evidence that adequate sampling of the tumor and the pancreas for pancreatectomies in patients with PDAC who received NAT has major implications on patient survival.

The recently published consensus paper from the PBPS on the pathologic examination of pancreatic specimens resected for PDAC after being treated with NAT recommended the entire submission of the tumor for cases with a tumor size of ≤2.0 cm and generous sampling (≥2 sections per cm of the tumor) for tumors that are >2.0 cm [12]. In this study, we demonstrated that ESOT was associated with better OS in patients with ypT0/T1 and ypN0 tumors. However, we did not observe significant associations between ESOT and either DFS or OS in patients with ypT2/ypT3 or positive lymph nodes. Our results support the recommendations from the PBPS that the entire tumor should be submitted for histologic examination if the tumor is 2.0 cm or less.

The consensus paper from the PBPS also recommended that the pancreas, peripancreatic soft tissue, common bile duct, ampulla of Vater, and duodenum adjacent to the pancreas should be entirely submitted or rigorously and extensively sampled for histologic examination to rule out microscopic foci of residual carcinoma if a pancreatectomy specimen for PDAC after NAT has no grossly identifiable tumor or has no viable residual carcinoma in the initially submitted sections [12]. This approach often identifies microscopic foci of residual carcinoma in the pancreas or adjacent tissue, which would be otherwise missed using representative sampling approach, and correctly classifies the ypT stage and tumor response grading. In this study, we demonstrated that ESOP was associated with better OS in patients with PDAC who had complete or near-complete pathologic response (MD Anderson grade 0 or 1). In addition, we demonstrated that ESOP was associated with better DFS and OS in patients with ypT0/T1 tumors. Interestingly, we also observed a significant association between ESOP with better DFS and OS in patients with ypN0 disease. However, no significant differences in either DFS or OS were observed between the group with ESOP and those without ESOP in patients with MD Anderson grade 2, CAP grade 2, or CAP grade 3 responses and in patients with ypT2/ypT3 or patients with positive lymph nodes. Our results support the approach of the entire submission or rigorous and extensive sampling of the pancreas, peripancreatic soft tissue, common bile duct, ampulla of Vater, and duodenum adjacent to the pancreas in patients with PDAC who have a complete pathologic response or a minimal amount of residual tumor (MD Anderson grade 0 or 1 response). Given the concerns of the cost and the time required for the pathologist to review the additional sections, the decision for the entire submission of the pancreas, peripancreatic tissue, common bile duct, ampulla of Vater, and duodenum adjacent to the pancreas is best made judiciously by the pathologist who is reviewing the specimen.

As expected, we found a significantly higher total number of lymph nodes in the PD specimens from the group with ESOP in our study. An adequate number of lymph nodes is critical for the accurate classification of the ypN stage, which has been shown to be one of the most important prognosticators of patients with PDAC [10,30,31,32].

The major limitations of this study are as follows: (1) this was a retrospective study at a single tertiary cancer center, which may have introduced potential selection bias into our study population. (2) At our institution, the entire submission of the pancreas was performed mainly due to the lack of a grossly identifiable tumor or initial representative sections with no or a minimal amount of residual carcinoma. In this retrospective study, however, it was challenging to determine the frequency with which ESOP and ESOT were performed intentionally during initial tissue sampling or as a result of missed carcinoma in the initial sampling in a two-stage approach. It is not surprising that the group with the entire submission of the pancreas had a smaller tumor size and a better pathology response, as we demonstrated in this study, which could have led to a potential bias in our survival analyses. However, the results of our survival analyses in different subgroups, including patients with complete or near-complete pathologic response (MD Anderson grade 0 or 1), ypT0/T1, and ypN0, consistently showed that ESOP was associated with better survival. More importantly, our results from multivariate Cox regression analyses demonstrated that ESOP remains an independent prognosticator of OS survival after adjusting ypT, ypN, tumor response grading, margin status, and tumor differentiation. Our study is the first large study to systemically examine the association of ESOP and ESOT with the clinicopathologic parameters and survival in patients with PDAC who underwent pancreatectomy after receiving NAT.

It should be noted that sampling (ESOP or ESOT) itself does not directly impact patient survival. However, inadequate sampling can lead to the underestimation of tumor size, residual viable tumor, lymphovascular and perineural invasion, and lymph-node metastasis, especially in patients with a near-complete pathologic response or a minimal amount of residual tumor. The inaccurate pathologic evaluation of these parameters can significantly affect survival and may explain the observed association between ESOP or ESOT and survival in our study.

## 5. Conclusions

Our study demonstrated for the first time that ESOP and ESOT are associated with ypT, perineural invasion, and tumor response grading. In addition, ESOP was also associated with the ypN stage, tumor differentiation, and lymphovascular invasion. However, since ESOP and ESOT were primarily conducted for cases with no grossly identifiable tumor or minimal residual carcinoma in initial sections, potential bias cannot be excluded. More importantly, we demonstrated that ESOP and ESOT were associated with less frequent recurrence/metastasis and better survival and were independent prognostic factors for OS according to multivariate survival analyses. Therefore, an accurate pathologic evaluation using ESOP and ESOT is important for the prognosis of PDAC patients with a complete or near-complete pathologic response and a ypT0/ypT1 tumor after NAT. 

## Figures and Tables

**Table 1:** Correlations between the entire submission of the tumor and the pancreas with clinicopathologic features in pancreaticoduodenectomy specimens after neoadjuvant therapy.

Characteristics	ESOT		*p*	ESOP		*p*
	Yes (n = 333)	No (n = 294)		Yes (n = 101)	No (n = 526)	
**Age (Years, Mean ± SD)**	63.9 ± 9.9	63.5 ± 9.0	0.57	64.2 ± 9.2	63.6 ± 9.7	0.56
Sex			0.96			0.27
Female	150	133		51	232	
Male	183	161		50	294	
Differentiation			0.56			**0.02**
Well/moderate	213	181		74	320	
Poor	120	113		27	206	
ypT *			**<0.001**			**<0.001**
ypT0	17	0		14	3	
ypT1	122	83		52	153	
ypT2	146	179		20	305	
ypT3	48	32		15	65	
ypN stage *			0.46			**0.001**
ypN0	156	128		63	221	
ypN1	111	112		26	197	
ypN2	66	54		12	108	
Lymphovascular invasion			0.34			**0.009**
No	170	138		62	246	
Yes	163	156		39	280	
Perineural invasion			**<0.001**			**<0.001**
No	97	49		47	99	
Yes	236	245		54	427	
CAP TRG			**<0.001**			**<0.001**
Score 0	17	0		14	3	
Score 1	53	22		27	48	
Score 2	169	161		43	287	
Score 3	94	111		17	188	
MD Anderson TRG			**<0.001**			**<0.001**
Grade 0	17	0		14	3	
Grade 1	53	22		27	48	
Grade 2	263	272		60	475	
Margin status			0.82			0.45
Negative	282	251		89	444	
Positive	51	43		12	82	
Recurrence/metastasis			**<0.001**			**0.03**
No	115	81		42	154	
Local recurrence	54	55		11	98	
Distant metastasis	164	158		48	274	

* According to AJCC 8th edition; ESOT, entire submission of the tumor; ESOP, entire submission of the pancreas; TRG: tumor response grading; CAP: College of American Pathologists.

**Table 2:** Univariate Cox regression analysis of disease-free survival and overall survival.

		Disease-Free Survival		Overall Survival	
Characteristics (N = 627)	N	HR (95% CI)	*p*	HR (95% CI)	*p*
Gender					
Female (ref)	283	1.00		1.00	
Male	344	1.06 (0.88–1.28)	0.56	1.09 (0.89–1.34)	0.38
Age	627	1.00 (0.98–1.01)	0.29	1.00 (0.99–1.01)	0.61
Tumor differentiation					
Well/Moderate (ref)	394	1.00		1.00	
Poor	233	1.33 (1.09–1.61)	**0.004**	1.32 (1.07–1.62)	**0.009**
Margin status					
Negative (ref)	533	1.00		1.00	
Positive	94	1.35 (1.04–1.74)	**0.02**	1.14 (0.85–1.53)	0.39
ypT			**0.003**		**0.01**
ypT0/T1 (ref)	222	1.00		1.00	
ypT2	325	1.36 (1.10–1.68)	**0.005**	1.35 (1.08–1.69)	**0.009**
ypT3	80	1.60 (1.18–2.17)	**0.003**	1.49 (1.06–2.08)	**0.02**
ypN stage			**<0.001**		**<0.001**
ypN0 (ref)	284	1.00		1.00	
ypN1	223	1.54 (1.24–1.91)	**<0.001**	1.62 (1.28–2.04)	**<0.001**
ypN2	120	2.52 (1.97–3.23)	**<0.001**	2.48 (1.90–3.23)	**<0.001**
MDA TRG					
Grade 0/1 (ref)	92	1.00		1.00	
Grade 2	535	1.94 (1.44–2.63)	**<0.001**	2.25 (1.61–3.15)	**<0.001**
Entire submission of tumor					
No (ref)	294	1.00		1.00	
Yes	333	0.85 (0.71–1.03)	0.10	0.74 (0.61–0.91)	**0.004**
Entire submission of pancreas					
No (ref)	526	1.00		1.00	
Yes	101	0.63 (0.48–0.83)	**0.001**	0.56 (0.41–0.76)	**<0.001**

MDA: MD Anderson Cancer Center; TRG: tumor response grading.

**Table 3:** Multivariate Cox regression analysis of disease-free survival and overall survival with the entire submission of the tumor.

		Disease-Free Survival		Overall Survival	
Characteristics (N = 627)	N	HR (95% CI)	*p*	HR (95% CI)	*p*
Tumor differentiation					
Well/moderate (ref)	394	1.00		1.00	
Poor	233	1.27 (1.05–1.54)	**0.02**	1.28 (1.04–1.58)	**0.02**
Margin status					
Negative (ref)	533	1.00		1.00	
Positive	94	1.09 (0.83–1.42)	0.53	0.88 (0.65–1.19)	0.88
ypT			0.73		0.84
ypT0/T1 (ref)	222	1.00		1.00	
ypT2	325	1.03 (0.82–1.29)	0.81	0.97 (0.77–1.24)	0.83
ypT3	80	1.14 (0.82–1.58)	0.44	1.07 (0.75–1.53)	0.70
ypN stage			**<0.001**		**<0.001**
ypN0 (ref)	284	1.00		1.00	
ypN1	223	1.43 (1.15–1.78)	**0.001**	1.48 (1.17–1.87)	**0.001**
ypN2	120	2.32 (1.81–2.99)	**<0.001**	2.32 (1.77–3.03)	**<0.001**
MDA TRG					
Grade 0/1 (ref)	92	1.00		1.00	
Grade 2	535	1.66 (1.22–2.25)	**0.001**	1.79 (1.26–2.52)	**0.001**
Entire submission of tumor					
No (ref)	294	1.00		1.00	
Yes	333	0.89 (0.73–1.08)	0.23	0.80 (0.65–0.98)	**0.03**

MDA: MD Anderson Cancer Center; TRG: Tumor response grading.

**Table 4:** Multivariate Cox Regression Analysis of Disease-free Survival and Overall Survival with Entire Submission of Pancreas.

		Disease-Free Survival		Overall Survival	
Characteristics	N	HR (95% CI)	*p*	HR (95% CI)	*p*
Tumor differentiation					
Well/moderate (ref)	394	1.00		1.00	
Poor	233	1.26 (1.04–1.54)	**0.02**	1.27 (1.03–1.56)	**0.02**
Margin status					
Negative (ref)	533	1.00		1.00	
Positive	94	1.09 (0.83–1.42)	0.54	0.88 (0.65–1.17)	0.41
ypT			0.74		0.79
ypT0/T1 (ref)	222	1.00		1.00	
ypT2	325	1.00 (0.80–1.25)	0.99	0.95 (0.75–1.21)	0.70
ypT3	80	1.12 (0.81–1.55)	0.49	1.06 (0.74–1.51)	0.75
ypN stage			**<0.001**		**<0.001**
ypN0 (ref)	284	1.00		1.00	
ypN1	223	1.42 (1.14–1.77)	**0.002**	1.47 (1.17–1.86)	**0.001**
ypN2	120	2.31 (1.79–2.97)	**<0.001**	2.24 (1.71–2.93)	**<0.001**
MDA TRG					
Grade 0/1 (ref)	92	1.00		1.00	
Grade 2	535	1.53 (1.12–2.10)	**0.008**	1.73 (1.21–2.45)	**0.002**
Entire pancreas submitted					
No (ref)	526	1.00		1.00	
Yes	101	0.77 (0.58–1.02)	0.07	**0.72 (0.53–0.99)**	**0.04**

MDA: MD Anderson Cancer Center; TRG: tumor response grading.

## Data Availability

The data are unavailable due to privacy or ethical restrictions.
